# Spatial organization of dendritic cells within tumor draining lymph nodes impacts clinical outcome in breast cancer patients

**DOI:** 10.1186/1479-5876-11-242

**Published:** 2013-10-02

**Authors:** Andrew Y Chang, Nupur Bhattacharya, Jian Mu, A Francesca Setiadi, Valeria Carcamo-Cavazos, Gerald H Lee, Diana L Simons, Sina Yadegarynia, Kaveh Hemati, Adam Kapelner, Zheng Ming, David N Krag, Erich J Schwartz, Danny Z Chen, Peter P Lee

**Affiliations:** 1 Department of Medicine, Stanford University, 269 Campus Drive, 94305 Stanford, CA, USA; 2 Department of Computer Science and Engineering, University of Notre Dame, 384 Fitzpatrick Hall, 46556 Notre Dame, IN, USA; 3 Department of Statistics, The Wharton School of the University of Pennsylvania, 400 Jon M. Huntsman Hall, 3730 Walnut Street, 19104 Philadelphia, PA, USA; 4 Department of Anesthesia, School of Medicine, Stanford University, 300 Pasteur Drive, Room H3580, 94305 Stanford, CA, USA; 5 University of Vermont College of Medicine, E309C Given Building, 89 Beaumont Avenue, 05405 Burlington, VT, USA; 6 Department of Pathology, Stanford University, 300 Pasteur Drive, Lane 235, 94305 Stanford, CA, USA; 7 Cancer Immunotherapeutics & Tumor Immunology, City of Hope and Beckman Research Institute, 1500 East Duarte Road, 91010 Duarte, CA, USA

**Keywords:** Breast cancer, Immune cells, Dendritic cells, Clustering, Spatial organization, Lymph nodes

## Abstract

**Background:**

Dendritic cells (DCs) are important mediators of anti-tumor immune responses. We hypothesized that an in-depth analysis of dendritic cells and their spatial relationships to each other as well as to other immune cells within tumor draining lymph nodes (TDLNs) could provide a better understanding of immune function and dysregulation in cancer.

**Methods:**

We analyzed immune cells within TDLNs from 59 breast cancer patients with at least 5 years of clinical follow-up using immunohistochemical staining with a novel quantitative image analysis system. We developed algorithms to analyze spatial distribution patterns of immune cells in cancer versus healthy intra-mammary lymph nodes (HLNs) to derive information about possible mechanisms underlying immune-dysregulation in breast cancer. We used the non-parametric Mann–Whitney test for inter-group comparisons, Wilcoxon Matched-Pairs Signed Ranks test for intra-group comparisons and log-rank (Mantel-Cox) test for Kaplan Maier analyses.

**Results:**

Degree of clustering of DCs (in terms of spatial proximity of the cells to each other) was reduced in TDLNs compared to HLNs. While there were more numerous DC clusters in TDLNs compared to HLNs,DC clusters within TDLNs tended to have fewer member DCs and also consisted of fewer cells displaying the DC maturity marker CD83. The average number of T cells within a standardized radius of a clustered DC was increased compared to that of an unclustered DC, suggesting that DC clustering was associated with T cell interaction. Furthermore, the number of T cells within the radius of a clustered DC was reduced in tumor-positive TDLNs compared to HLNs. Importantly, clinical outcome analysis revealed that DC clustering in tumor-positive TDLNs correlated with the duration of disease-free survival in breast cancer patients.

**Conclusions:**

These findings are the first to describe the spatial organization of DCs within TDLNs and their association with survival outcome. In addition, we characterized specific changes in number, size, maturity, and T cell co-localization of such clusters. Strategies to enhance DC function in-vivo, including maturation and clustering, may provide additional tools for developing more efficacious DC cancer vaccines.

## Background

There is growing evidence that the immune system naturally responds against cancer. In a series of 1919 cases of primary ductal and lobular infiltrating breast carcinomas, a strong positive correlation was found between survival rates and the presence of lymphocytes at the tumor site [1]. More recently, gene expression analysis of 1781 primary breast cancer samples showed that the presence of a functional T cell metagene signature predicted a favorable prognosis in ER-negative and HER2-positive breast cancers [2]. In yet another study [3], multivariate analysis revealed that infiltration of primary breast tumors with mature dendritic cells (DCs) had independent favorable prognostic relevance in breast carcinomas.

An anti-tumor response is elicited when DCs present tumor antigens to T cells leading to activation and proliferation of cancer-specific T cells. Over the past decade, there has been considerable effort made to characterize DC populations in patients with cancer. Peripheral blood DC numbers are shown to be altered in patients with head and neck, breast, colorectal, gastric, lung, cervical, endometrial, and renal cell carcinomas [4-6]. Low numbers of DCs within the tumor correlated with poor clinical outcome in several different cancers including breast, colorectal, gastric, esophageal, thyroid, and bladder transitional cell carcinomas [7-9].

Beyond numbers, the maturation status of DCs also plays an important role in determining the nature of an immunologic response. Mature DCs are capable of eliciting an effective “immunogenic” response. While immature DCs can process and present antigen in the context of MHC class I molecules, this results in the induction of a “tolerogenic” response [10]. There is strong evidence that cancer affects DC maturation and differentiation. In primary breast tumor masses, DCs interspersed within the tumor bed usually lack maturation markers [11,12]. Breast tumor-derived factors such as prostanoids [13], macrophage inhibitory protein 3 alpha [11], and spermine [14] have been found to correlate with decreased DC maturation.

One of the pivotal locations to examine immune-tumor interactions is the tumor-draining lymph node (TDLN). It is the site where tumor antigens are typically first presented to the immune system and a critical initial decision between immune activation and tolerance is made. In a prior study, our group found significant decreases in immune cell populations within breast cancer non-sentinel lymph nodes (NSLNs), specifically in CD4^+^ T cells and CD1a^+^ DCs, and discovered that these changes strongly correlated with clinical outcome [15].

The aims of the present study were to examine the spatial distribution of immune cells within TDLNs as compared to HLNs, determine if these spatial patterns are associated with clinical outcome, and apply quantitative methods to characterize the spatial organization of these cells in vivo. To this end, we hypothesized that the spatial distribution of immune cells within TDLNs could also be altered by cancer and could provide additional information about the underlying tumor-immune interplay. One such spatial phenomenon described in the literature is that of “clustering” behavior seen in the DC population. Several studies have detailed aggregations of DCs, both in-vitro and in-vivo, noting functional properties associated with cluster size and maturation levels [16-18]. Alterations in DC clustering were associated with autoimmune diseases in murine models and humans [16-18]. We thus focused our analyses on the spatial organization of immature DCs, mature DCs, and T cells within breast cancer TDLNs and compared them to cell distributions in healthy lymph nodes (HLNs). We found that DCs in HLNs tend to aggregate in large clusters of mature cells, whereas DCs in TDLNs tend to remain either unclustered or form smaller clusters with fewer mature cells. This clustering behavior appears to be associated with an increased number of proximal T cells co-localized to a clustered DC compared to an unclustered DC. Importantly, a higher degree of DC clustering within TDLNs correlated with better clinical outcome in breast cancer patients.

## Methods

### Study patients

59 breast cancer patients aged 32–80 years, treated at Stanford University Medical Center between 1995 and 2011 were included in this study. For controls, seven intramammary lymph nodes were obtained from healthy prophylactic mastectomy and breast reduction patients aged 29–48 years old (median: 40) treated at Stanford University Medical Center between 2001 and 2007. These tissue samples were acquired from the Stanford Pathology Department archive as coded specimens under a protocol approved of by the Stanford University Medical Center Institutional Review Board (IRB). In addition, 6 breast cancer patients, aged 37–54, treated between 1995 and 2009 at the University of Vermont Fletcher Allen Breast Care Center were included in the study. At the time of tissue acquisition by lymph node biopsy, all participants were untreated and were without previous recorded history of cancer or autoimmune disease. Initial diagnoses were made by needle aspiration or core biopsy for most cases. Final diagnoses were confirmed by pathologic evaluation of the excised tissue specimen. Following appropriate surgical intervention, all patients received adjuvant radiation or chemotherapy per recommendations by their primary treatment physicians. Minimal clinical follow up for all patients was 5 years after diagnosis unless patients relapsed within the first 5 years. A privacy notice informed patients that their medical records could be used for scientific research without their authorization, upon IRB approval. The confidentiality of patients’ identifying information was protected at all times, with data gathered during the study not to influence the treatment of the subjects and their well being.

### Tissue preparation and immunostaining

Tissue samples were acquired as 3μm-thick serial cuts via microtome from formalin-fixed paraffin-embedded blocks. Antigen retrieval was then achieved using Diva Decloaker (Biocare Medical, Concord, CA). Two multi-color immunohistochemistry (IHC) panels were devised to simultaneously visualize the relative locations of immature DCs, mature DCs, T cells, tumor cells, and the nuclei of other cells not specifically stained for by antibodies. IHC was performed on serially sectioned tissue specimens.

Breast cancer cells were identified by pan-cytokeratins AE1/AE3 positivity, immature DCs by CD1a positivity and CD83 negativity, mature DCs by CD83 positivity, and T cells by CD3 positivity. Primary antibodies utilized included pan-cytokeratin (mouse monoclonal, clones AE1/AE3, 1:200 from Biocare), CD1a (mouse monoclonal, clone CD1a007, 1:50 from Biocare), CD83 (mouse monoclonal, clone HB15a, 1:4000 from Beckman Coulter, Marseille, France), and CD20/CD3 (mouse monoclonal, clone L26/rabbit monoclonal, clone SP7 from Biocare Medical) double stain cocktail (although analysis of B cells was not included in this study). Secondary antibodies used were Mach 2 Mouse AP-Polymer Detection System (Biocare) for CD1a and pan-cytokeratins, Mach 2 Mouse HRP (Biocare) for CD83, and Double Stain 2 (anti-mouse HRP, anti-rabbit ALP cocktail, Biocare) for CD3+CD20. Chromogens utilized were NBT-BCIP (Dako, Carpinteria, CA) for pan-cytokeratins, Vulcan Fast Red (Biocare) for CD1a, DAB (Biocare) for CD83, Ferangi Blue (Biocare) for CD3. Denaturing solution (Biocare) was used between each stain to clear residual reagent left from the previous sequence. Each slide was also counterstained with hematoxylin (Biocare) to visualize all nuclei. Optimization of antibody concentration and incubation time was performed using tonsil control tissue.

### Imaging

Processed and stained slides were imaged using a custom-built automated high-resolution multispectral imaging system (CRi Vectra™, Woburn, MA). This setup is capable of capturing digital images at 200×, outputting data sets of hundreds of Tagged Image Files (TIF) which can be stitched together to recreate a whole tissue section. Vectra™ images each subject at increments of 12 wavelengths between 420 to 720nm, then utilizing user-directed examples of each chromogen from a training set to spectrally un-mix the chromogen signatures into independent channels.

### Image analysis

#### Cell classification

The files created by Vectra™ were loaded into GemIdent, a custom statistical image segmentation program using machine-learning to identify/classify distinct cell types and quantify them [19]. The software accomplishes this task by using iterative trainings by an operator to build a classification library. GemIdent is then able to apply the user-defined examples in its library to automatically classify all cells found in a loaded data set. Maps are then constructed by the program, indicating the type, number, and Cartesian (x, y) coordinates of identified cells on each slide. Factors taken into account by the system include color, size, and morphological features of marked cells. The number of training examples needed depends on the integrity of the tissue and quality of IHC staining, with typical tissue sections requiring between 50 and 200 examples per slide [20].

#### Defining DC clusters

To quantitatively analyze the spatial patterns of DCs identified by GemIdent, we applied the density-based clustering (DBC) algorithm of Chen et al. [21] to define and identify “clusters” of DCs. Clustering, by definition, is a classification of an input set S of objects into groups (i.e., subsets of S) based on certain criteria, such that the objects in the same cluster are more similar to each other according to the specified partitioning criteria than to objects in different clusters. The DBC algorithm groups DCs that are close to each other in Euclidean distance into clusters while ignoring “isolated” DCs (i.e., the DCs that do not belong to any cluster), using an approach which assesses the density of nearby DCs in the neighborhood of each DC. The clusters determined by the DBC algorithm are mutually disjoint (i.e., no two different clusters share any common element).

The main concept of the DBC algorithm is defined as follows:

Input: A point set S.

Output: A collection of clusters C_1_, C_2_, …, C_k_ of S.

Parameters: R (a radius for specifying a circular region for the neighborhood) and D (for the density of DCs within the neighborhood).

1. For each point p ∈ S, count the number n(p) of points of S inside the circle C(p) of radius R centered at p; if the number n(p) ≥ D, then all the points of S ∩ C(p) are said to be clustered, and they are assigned the same cluster label;

2. For any two clusters C_i_ and C_j_, if C_i_ ∩ C_j_ ≠ ϕ, then group C_i_ and C_j_ into the same cluster;

3. Any unclustered point of S is considered as “noise” and is discarded.

For two chosen parameters R and D, if the circle C of radius R centered at any DC contains at least D DCs, then all DCs in the circle C are considered part of a cluster. To prevent double counting, if two clusters determined by the algorithm share any common DCs, then they are merged into one cluster. If any DC does not belong to any cluster at the end of the clustering process, then it is considered as isolated or “unclustered”. In studying the properties and patterns of DC clusters, isolated DCs are not taken into account (Additional file 1).

Clustering results were computed using different pairs of R and D values, comparing the results against a training set of six randomly-selected tissue slides containing DC aggregations in parafollicular zones as identified by a biologist. The pair of parameters that yielded the best match was selected. Based on the optimization set, a circle of radius R = 250 (in pixel units of GemIdent output images) and density value D = 5 were chosen.

#### DC-T interactions

DCs interact with T cells in lymph nodes to initiate cell-mediated immune responses. Two-photon studies have described T cells moving in a looping, “orbital” fashion within lymph nodes, making physical contacts with DCs [22]. A T cell often needs to make multiple such connections with DCs at their dendritic projections in order to find an appropriate antigenic partner. To define a DC to T cell “contact”, we set a co-localization radius of a DC and T cell to be 100 pixels, which is approximately the diameter of a DC (including its dendrites) in the output images of our digital histology system. Since a T cell must enter a theoretical influence radius of a DC in order to make possible physical contact with it, we defined any T cell within 100 pixels of its nearest DC as being associated with that DC. All such T cells within this radius of a clustered DC would be defined as “clustered”. Obviously, those not within 100 pixels of a DC were considered unassociated. Note that our model ignores other methods of influence DCs exert on T cells (e.g., soluble attractive factors), considering only the potential for physical associations within the 100-pixel range.

### Statistical analysis

The non-parametric Mann–Whitney Test was applied for inter-group comparisons and the Wilcoxon Matched-Pairs Signed Ranks test was utilized for intra-group comparisons. To compare the number of years of disease-free survival for patients with different DC parameters, only those patients who had a minimum follow-up period of 5 years from the date of surgery (range: 5.5 years to 12 years) and had no diagnosed concurrent cancers or neoplastic processes at the time of breast cancer diagnosis were analyzed. Furthermore, only patients whose total percentage of DCs (out of all counted cells in a lymph node) was in the center 90% range (i.e. above the 5^th^ percentile and below the 95^th^ percentile) were considered for the survival analysis. For each of the DC parameters considered, patients were divided into two groups based on the value of the parameter being considered. To avoid introducing spurious findings due to biased selection of thresholds, the median value was always chosen to divide the patients into two equally-sized groups. Kaplan-Meier curves were plotted to visually inspect the survival difference between the two groups and the log-rank (Mantel-Cox) test was applied to determine the statistical significance of the difference. The GraphPad Prism 5.0 (La Jolla, CA) software package was used for all our statistical analyses. A p-value <0.05 was considered a statistically significant difference in these calculations.

## Results

### Patient, tumor, and lymph node attributes

Clinicopathologic characteristics of the study patients and tissue samples, including number, age, tumor size, grade/stage, molecular subtype, and steroid receptor status are shown in Table 1. Nodes were segregated into three groups: 50 were tumor-free non-sentinel lymph nodes (NSLN-), 22 were tumor-invaded non-sentinel lymph nodes (NSLN+), and 7 were healthy control nodes (HLNs). Of the breast cancer patient lymph nodes, 7 NSLN- and NSLN+ were pairs from the same patients. All patients had tumor-positive sentinel nodes on dissection.

**Table 1:** Clinical pathological characteristics associated with NSLNs

	**Tumor free NSLNs (NSLN-)**	**Tumor invaded NSLNs (NSLN+)**
**Number of patients**	n=50	n=22
**Median age of patients (years)**	median : 47 range: 32-80	median: 54 range: 32-76
**Primary tumor size (cm)**	median: 2.05 range: 0.30-7.00	median: 2.15 range: 0.80-7.00
**Stage**		
I	5/50 (10%)	…
IIA	20/50 (40%)	4/22 (18.2%)
IIB	13/50 (26%)	4/22 (18.2%)
III	7/50 (14%)	9/22 (41%)
Unknown	5/50 (10%)	5/22 (22.7%)
**Grade**		
I	8/50 (16%)	…
II	30/50 (60%)	8/22 (36.3%)
III	9/50 (18%)	9/22 (41%)
Unknown	3/50 (6%)	5/22 (22.7%)
**Molecular subtype**		
Luminal A	23/50 (46%)	8/22 (36.3%)
Luminal B	10/50 (20%)	3/22 (13.6%)
HER2	2/50 (4%)	4/22 (18.2%)
Basal	3/50 (6%)	2/22 (9%)
Unknown	12/50 (24%)	5/22 (22.7%)
**ER status**		
Positive	41/50 (82%)	15/22 (68.1%)
Negative	6/50 (12%)	7/22 (31.9%)
Unknown	3/50 (6%)	…
**PR status**		
Positive	38/50 (76%)	13/22 (59%)
Negative	9/50 (18%)	9/22 (41%)
Unknown	3/50 (6%)	…
**HER2/neu expression**		
Positive	12/50 (24%)	7/22 (31.8%)
Negative	26/50 (52%)	10/22 (45.5%)
Unknown	12/50 (24%)	5/22 (22.7%)

### DC number, maturity, and spatial organization are altered in breast cancer TDLNs compared to HLNs

**Figure 1:**
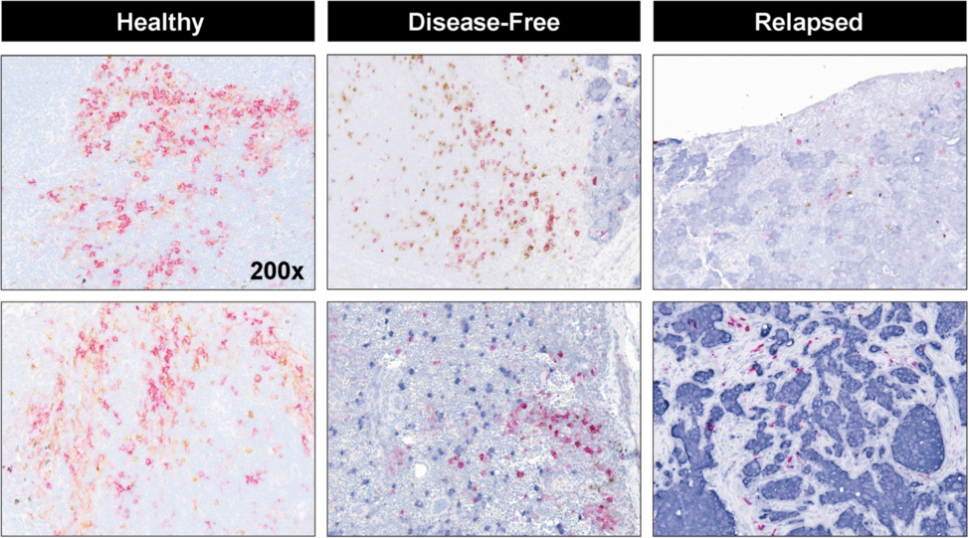
**Immunohistochemistry-Stained Images of DC Clusters.** Immunohistochemistry stained images of DC (dendritic cell) clusters from two each healthy, disease-free and relapsed patients. Stains include: Magenta (CD1a, Immature DCs), Brown (CD83, Mature DCs), Blue (Hematoxylin, Non-DC cells). The dark purple cells are pan-cytokeratin-stained tumor cells. Note the increased number and density of DCs in control nodes. All images were taken at 200× resolution.

**Figure 2:**
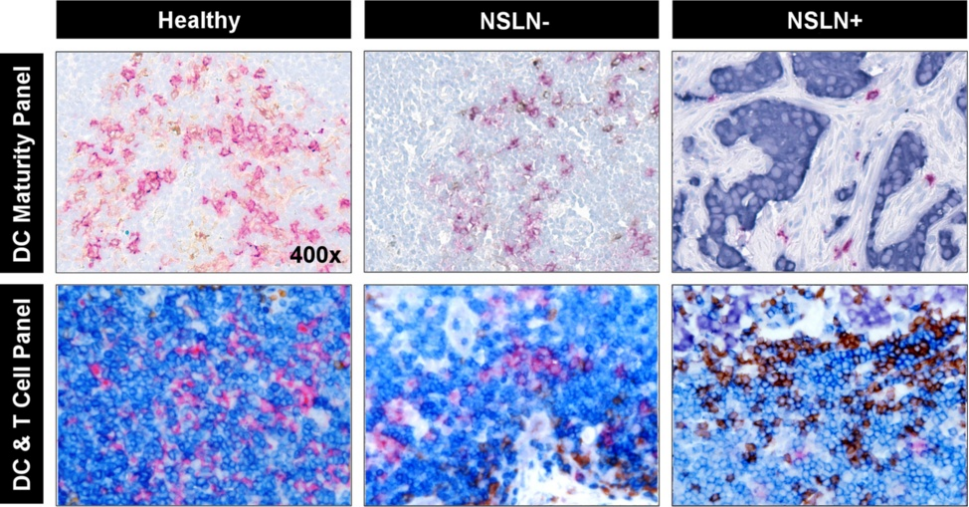
**High-resolution IHC Images of DC Clusters in two staining panels.** Immunohistochemistry stained images of DC clusters from healthy, NSLN- and NSLN+ patient samples, serially stained in both a DC maturity assessment panel and a T cell colocalization panel. Stains for the maturity panel include: Red (CD1a, Immature DCs), Brown (CD83, Mature DCs), Blue (Hematoxylin, Non-DC cells). Stains for the T cell colocalization panel include: Magenta (CD1a, Immature DCs), Dark Blue (CD3, T cells), Brown (CD20, B cells), Light Blue (Hematoxylin, other cells). The dark purple cells in both panels are pan-cytokeratin-stained tumor cells. All images were taken at 400× resolution.

**Figure 3:**
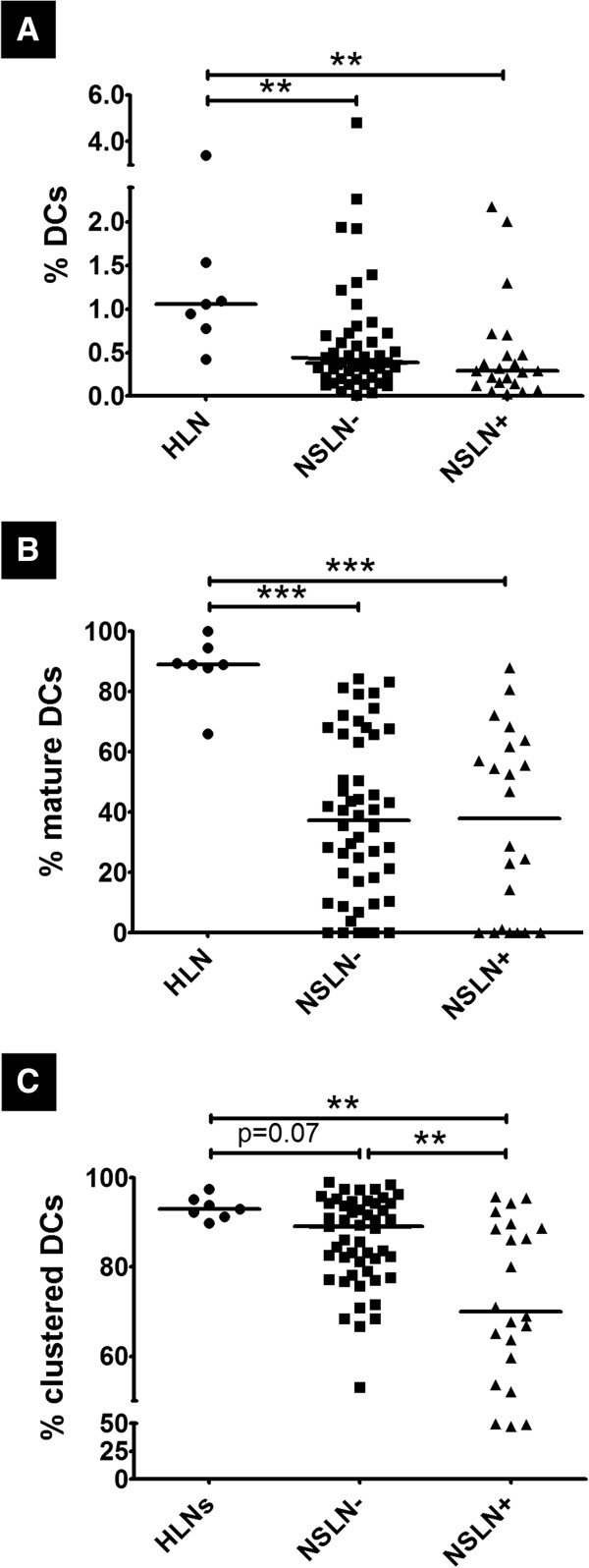
**DC number, maturity, and organization are altered in breast cancer TDLNs compared to HLNs. (A)** Percentage dendritic cells (DCs) out of all T cells, DCs, and other hematoxylin-stained cells identified in a node (%DCs), **(B)** Percentage of DCs expressing CD83 out of all DCs expressing CD83 and/or CD1a (% mature DCs) and **(C)** Percentage of DCs that formed clusters out of all DCs in a node (% clustered DCs) were calculated in sections of healthy lymph nodes: HLN (n=7), Tumor-free non-sentinel lymph nodes: NSLN- (n=50) and Tumor-involved non-sentinel lymph nodes NSLN+ (n=22). ** denotes p<0.01 and *** denotes p<0.001.

To determine how DCs in TDLNs may differ from those of HLNs, we assessed alterations in DC number and maturity between these groups. First, all stained slides were microscopically examined, and it was observed that semi-quantitatively, HLNs had a greater number of DCs in each section than NSLN- and NSLN+. The pattern was seen between HLNs and the TDLNs of breast cancer patients who remained disease-free or relapsed during a five-year follow-up period post-treatment. HLN DCs appeared to form larger, more densely populated “clusters” than their TDLN counterparts (Figures 1 and 2). Given these qualitative differences, immune cell numbers and spatial distributions were analyzed quantitatively using a novel quantitative, spatial analysis imaging approach [21]. Since there are variations in size and cell number among lymph nodes, we used DCs as a percentage of total cells in a node (calculated as a sum of all T cells, DCs, and other hematoxylin-stained cells identified and counted by GemIdent) to account for this. Total DCs were counted as all cells in a node that expressed CD1a and/or CD83 (to account for both immature and mature DC populations). We found that DCs were significantly decreased in both NSLN- (median value: 0.38%, p<0.01) as well as NSLN+ (0.29%, p<0.01) compared to HLNs (1.06%, Figure 3A). The percentage of mature DCs in both NSLN- and NSLN+ (defined as the percentage of total DCs in each node that was CD83-expressing) was also significantly reduced compared to healthy controls (median value: 37.35%, p<0.001 and 37.89%, p<0.001 respectively vs. 89.00%) (Figure 3B). Moving beyond numbers of individual cell types, we hypothesized that spatial organization of DCs may also be altered. We quantified the clustering behavior of DCs using the DBC algorithm we developed. As with total overall DC number, we took into account variation in lymph node size and evaluated the degree of clustering in each node as the percentage of total DCs that were classified as in a cluster by our algorithm (Figure 3C). We observed a stepwise reduction in DC clustering, as a trend (p=0.07) towards decreased clustering in DCs from NSLN- (88.89%) compared to HLNs (93.05%), a significant difference in NSLN+ (69.99%) versus healthy nodes (p<0.01), and a significant reduction in NSLN+ versus NSLN- (p<0.01). Importantly, DC clustering was not merely a reflection of the percentage of DCs in a lymph node, as there was a significant reduction in DC clustering in NSLN+ nodes even though the percentage of DCs between NSLN+ and NSLN- nodes were similar (Figure 3A).

### DCs in TDLNs are organized into smaller, less mature clusters compared to HLNs

**Figure 4:**
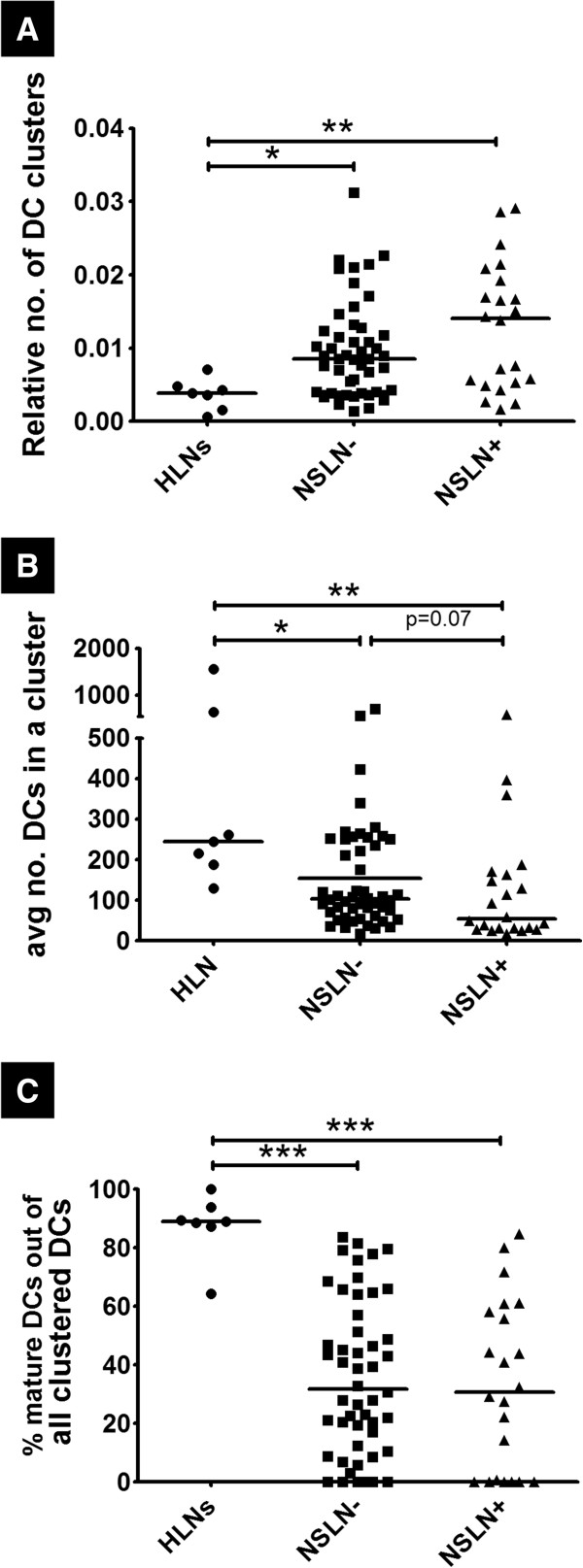
**DCs in TDLNs are organized into smaller, less mature clusters compared to HLNs. (A)** Number of DC clusters normalized to the total number of DCs in a node (relative number of DC clusters) **(B)** average size of a DC cluster in a node (calculated by taking an average of the number of DCs that make up individual DC clusters in a node) and **(C)** percentage of CD83 positive DCs of all clustered DCs in a node (% mature DCs out of all clustered DCs) were calculated in sections HLN (n=7), NSLN- (n=50) and NSLN+ (n=22). *denotes p<0.05, ** denotes p<0.01 and *** denotes p<0.001.

Alterations of DC clusters in the cancer state were examined by comparing the number of clusters, average number of DCs per cluster, and percentage of mature DCs per cluster between TDLNs and HLNs. First, the number of clusters in each node was computed, and to account for the lymph node size variation, this value was normalized to the total number of DCs in the node. We found that the relative number of clusters in each node was significantly increased in NSLN- (median value: 8.51×10^-3^ units, p<0.05) and NSLN+ (14.05×10^-3^ units, p<0.01) compared to HLNs (3.81×10^-3^ units, Figure 4A). However, DC clusters in TDLNs were found to contain fewer cells, as the mean number of DCs per cluster was significantly decreased in NSLN- (104 DCs, p<0.05) and NSLN+ (55 DCs, p<0.01) compared to healthy nodes (244 DCs, Figure 4B). This stepwise reduction was also observed in a trend found between NSLN- and NSLN+ (p=0.07). The maturation of the DCs in clusters was also altered in the nodes of cancer patients, with the percentage of mature DCs out of all clustered DCs in each node reduced significantly in NSLN- (31.77%, p<0.001) and NSLN+ (30.73% p<0.001) compared to HLNs (88.86%, Figure 4C).

### T cells surrounding a clustered DC is reduced in NSLN+ compared to HLNs

**Figure 5:**
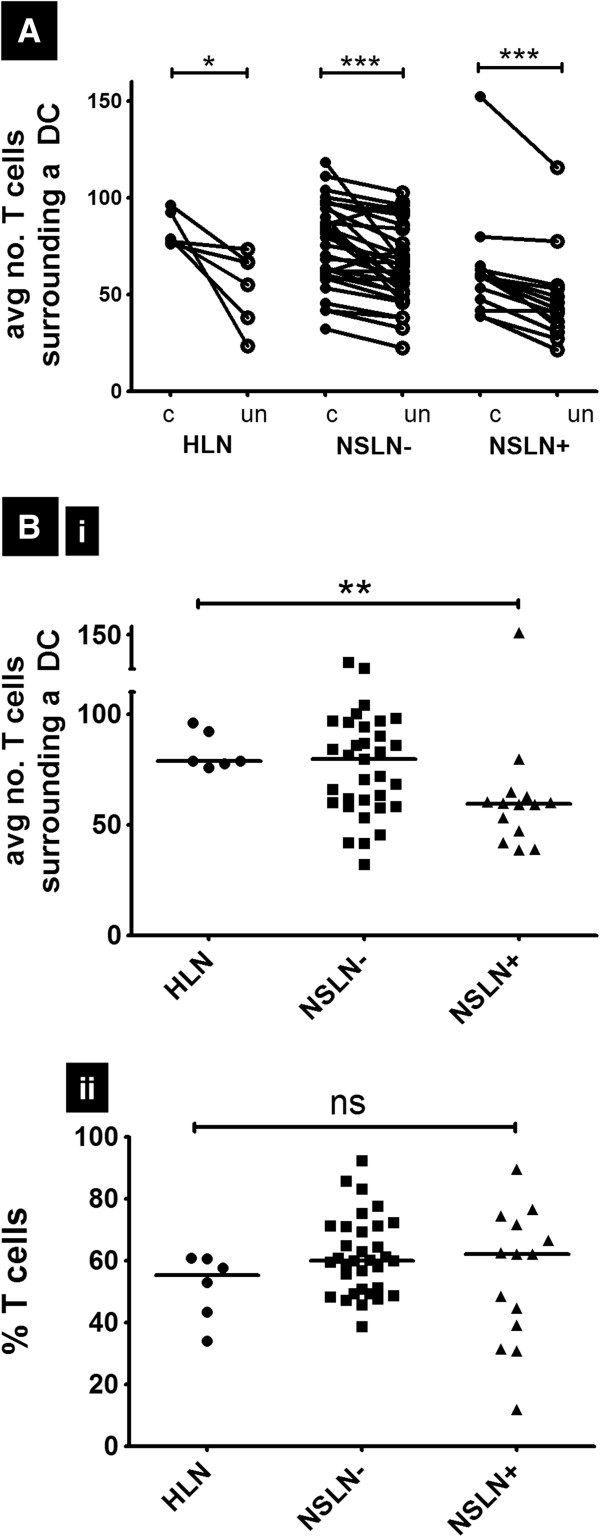
**T cells surrounding a clustered DC is reduced in NSLN+ compared to HLNs. (A)** Average number of T cells surrounding clustered DCs (c) versus unclustered DCs (un) (calculated using an algorithm mentioned in the Methods section) **(B)** (i) Average number of T cells surrounding a clustered DCs and (ii) Percentage T cells out of all T cells, DCs, and other hematoxylin-stained cells identified in a node (%T cells) were calculated in sections of HLN (n=6), NSLN- (n=33) and (n=14). ns denotes p>0.05, * denotes p<0.05 and *** denotes p<0.001.

To investigate if DC clustering is associated with T cell behavior, we developed a DC-T co-localization algorithm examining the proximity of T cells to DCs within a predetermined influence range. The mean number of T cells found within a 100-pixel radius of a DC was significantly reduced in unclustered DCs compared to clustered DCs (Figure 5A). This phenomenon was consistent within all our study lymph node populations: HLN (79 T cells surrounding clustered DCs vs. 61 T cells surrounding unclustered DCs, p<0.05), NSLN- (81 T cells surrounding clustered DCs vs.61 T cells surrounding unclustered DCs, p<0.001), and NSLN+ (60 T cells surrounding clustered DCs vs. 42 T cells surrounding unclustered DCs, p<0.001), which suggested that DCs are co-localized with T cells to a greater extent when organized in clusters. Furthermore, we found that there were significantly (p<0.01) fewer T cells localized within the radius of clustered DCs in NSLN+ (60 T cells) compared to healthy nodes (79 T cells, Figure 5Bi). To exclude the possibility that this finding may be simply a reflection of the reduced number of T cells in tumor-invaded lymph nodes, we also calculated T cells as a percentage of total cells in the nodes of each population. This showed that the percentage of total T cells in HLNs, NSLN-, and NSLN+ were not significantly different (Figure 5Bii), suggesting that the reduced T cells within the radius of clustered DCs in NSLN+ was not merely a reflection of fewer T cells in these nodes.

### DC clustering and maturation in NSLN+ nodes correlate with duration of disease-free survival

**Figure 6:**
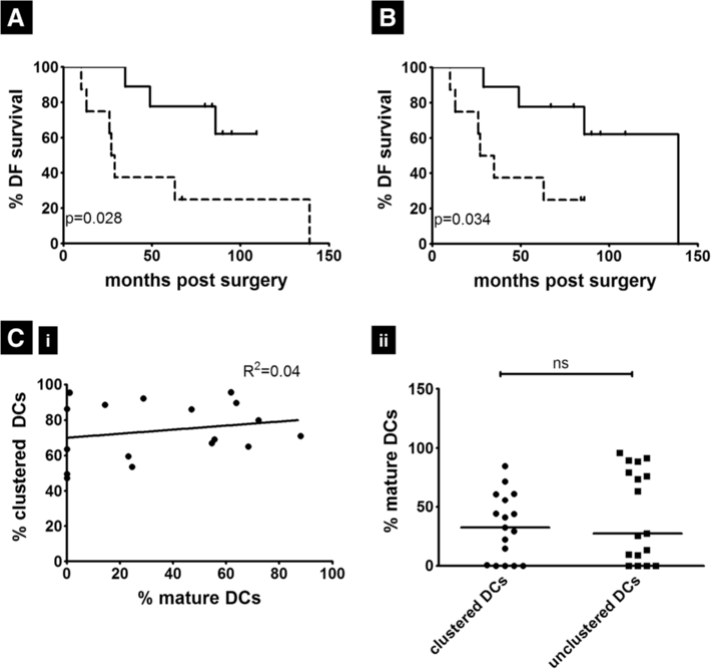
**DC clustering and maturation in NSLN+ nodes correlate with duration of disease-free survival.** Using the median value of **(A)** percentage mature DCs or **(B)** percentage clustered DCs per node as cutoff, patients having NSLN+ nodes (n=19) were divided into two groups (using exclusion criteria mentioned in the Methods section) with those above the median defined by the solid line and those below the median by the dotted line. Kaplan-Meier curves were plotted showing the difference in duration of disease free survival between the two groups. **(C)** (i) Linear regression analysis between %clustered DCs and %mature DCs in NSLN+ nodes (n=19) (ii) Percentage mature DCs were calculated among clustered as well as unclustered DCs in NSLN+ nodes (n=19) ns denotes p>0.05.

To determine if alterations in DC organization within TDLNs were associated with clinical outcome, survival analyses were performed to examine the relationships between DC parameters and patient disease-free remission. Using the median value of percent mature DCs per node to divide the patients into low (below median) and high (above median) groups, we found that the high group had a statistically significantly longer (p<0.028) duration of survival compared to the low group (Figure 6A). This difference was not observed to be statistically significant in NSLN- nodes. Similarly, we then grouped the patients into low (below median) and high (above median) groups based on the percentage of clustered DCs of all DCs in each node. There was a statistically significant (p=0.034) disease-free survival difference between the two groups (Figure 6B). DC clustering and maturation in NSLN+ nodes both correlated with duration of disease-free survival in our breast cancer patient population. To determine if clinical characteristics or medical treatment provided to groups above and below the median could account for the survival difference, we examined hormone receptor status, Her2/Neu expression, radiation therapy, and chemotherapy regimens of these cohorts. No significant differences were observed between either of the two comparison groups for those variables (Additional file 2, Additional file 3). Importantly, DC clustering was found to be independent of DC maturity status in the NSLN+ group (R^2^=0.04, p=0.416, Figure 6Ci). Furthermore, the percentage of mature DCs did not differ significantly between unclustered and clustered DC populations (p=0.425, Figure 6Cii). Therefore, DC clustering is not merely a function of maturation or vice versa.

## Discussion

DCs perform a central role in the initiation and regulation of anti-tumor immunity. Therefore, a number of studies have evaluated DC characteristics in the context of cancer. The majority of findings have shown that DC frequency, maturation status, and function are reduced in the cancer state. These findings are clinically relevant, as these were associated with a worse prognosis in patients [23]. These observations underlie the scientific rationale for devising immunotherapeutic strategies to enhance DC functionality in cancer [24]. A major milestone in DC based immunotherapy was the FDA approval of PROVENGE (sipuleucel-T; a dendritic cell-based vaccine for metastatic, hormone refractory prostate cancer). However, Phase III clinical trials showed only a modest improvement in median survival (by 4.1-months) in the sipuleucel-T group vs. the placebo group [25]. Therefore, there is substantial room for improvement. In order to achieve a long-lasting and effective anti-tumor response, it is important to recognize and develop immunotherapeutic strategies that address underlying tumor-derived immune dysfunction.

The immune status of TDLNs is emerging as an important factor in determining the development of subsequent immune responses to cancer. This present study extends our previous observation that a significant decrease in DC number in breast cancer NSLNs (compared to control lymph nodes) predicted clinical outcome [15]. Moving beyond numerical changes, we investigated another level of complexity in tumor induced immune dysfunction: the effect of cancer on the spatial organization of immune cells within TDLNs. We hypothesized that since lymph nodes represent specialized immune organs, spatial orientation and distribution of immune cells within the nodes could affect the generation of effective immune responses. This study comes at a time when there is a growing movement to incorporate the immune profiles of tumor tissues into the prognostic evaluation of patient pathology specimens [26]. The proposed “immune score” methodology includes not only the presence and number of immune cells, but also their general localization within tumors. Our findings provide new prognostic indicators from TDLN samples and insights into spatial distribution of immune cells in greater detail.

To this end, we developed a quantitative, spatial image analysis approach to histology. This begins with high dimension (4-color) immunohistochemistry stained tissue samples and imaging with an automated high-resolution, whole-section multi-spectral scanning system. The resulting output files were analyzed using a custom software suite (GemIdent), which utilizes user-driven machine learning to identify and determine the spatial localization of various indicated cell types. Lastly, we developed novel spatial analysis algorithms to quantify the spatial organization of tumor and immune cells within tissue. These methods have been evaluated as being both efficient and accurate when dealing with immunohistochemistry, whole-slide imaging, and cancer tissue samples [27,28]. Previous histological studies have obtained only qualitative or semi-quantitative results, since they base their findings on observing either a limited predetermined number of microscopic fields per tissue specimen or only the most densely populated cellular region of a lymph node. Our approach provides a comprehensive and quantitative understanding of the entire tissue section.

We used CD1a as a marker of immature DCs, CD83 as a marker for mature DCs, CD3 as a marker for T cells, and pan-cytokeratins as a marker for breast cancer cells. In human DCs, the cell surface expression of the CD83 protein is upregulated during the maturation process and has also been shown to play an important role in the regulation of DC-mediated immune responses [29]. CD83 expression in DCs within primary breast tumors was shown to correlate directly with clinical outcome [3]. CD1a has been used widely to identify myeloid DCs, and often together with S100, to define DC populations in breast tumor tissues and TDLNs [15,30-32]. There is evidence that CD1a may be involved in the presentation of tumor specific glycolipids to T cells, inducing CD1a-restricted tumor-specific T-cell responses [33].

A novel hypothesis of this study is that not only numerical changes, but also spatial organization of DCs, may impact immune function and clinical outcome in cancer. Using novel spatial analysis algorithms, we found that DCs in breast cancer NSLNs (especially in tumor-involved NSLNs) were either unclustered or were organized into smaller clusters with fewer mature DCs. In contrast, DCs in HLNs were greater in number and had bigger clusters predominantly composed of mature DCs. Importantly, the extent of DC clustering in NSLN+ nodes correlated with the duration of disease-free survival. In determining differential survival, this clustering behavior appears to be a factor independent of DC maturity status, major tumor characteristics, and major treatment regimens.

The phenomenon of DC clustering has been observed by several other groups, but these studies were largely qualitative in nature. Recently it was shown that during acute rejection of kidney transplants, mature myeloid DCs aggregate together in clusters, a behavior which was correlated with the loss of renal function [17]. These clusters were found in close proximity to T cells, suggesting that DC clustering was associated with the aggregation and activation of T cells with subsequent graft rejection [17]. In a murine model of inflammatory bowel disease, large intestinal DC aggregates were shown to participate in the generation of regulatory T cells (Tregs) [18]. Delemarre and colleagues carried out functional studies of DC clustering in rats, showing that clustering of rat splenic DCs (sDCs) led to an increase in DC maturation and increase in T cell stimulating capabilities in-vitro. Furthermore, they found that sDCs from biobreeding diabetes-prone rat (a rodent autoimmunity model) formed fewer and smaller clusters with modest levels of maturation [16].

To our knowledge, this is the first report on DC spatial organization in the context of human cancer. Prior studies on DC clustering suggested that 1) clustering is associated with DC maturation, and 2) clustering increases interactions of DCs with T cells. Interestingly, NSLNs that had a higher number of clustered DCs did not necessarily have a higher number of mature DCs and vice versa. This suggests that DC maturation and DC clustering could impact on clinical outcome in breast cancer patients independently. Furthermore, we found that the average number of T cells located around clustered DCs was significantly greater than the number of T cells around unclustered DCs in all NSLNs and HLNs analyzed. In addition, the average number of T cells surrounding clustered DCs was significantly reduced in tumor-invaded NSLNs compared to HLNs, even when the overall percentage of T cells per node was similar between the two groups. These findings suggest that either clustering makes DCs more effective at co-localizing with T cells or that T cell interaction is a key factor influencing cluster formation in DCs. Furthermore, DC clusters in tumor-involved nodes may have a reduced capacity to interact with and activate T cells compared to DC clusters in healthy nodes. Such phenomena are not discernible by eye and therefore would not have been appreciated without the novel quantitative approach we developed to quantify spatial distributions of cells.

The current study is limited by the lack of granularity in regards to the subsets of investigated immune cell types. A detailed investigation of different T cells (e.g., CD4^+^ T cells, CD8^+^ T cells, regulatory T cells) and DC subsets (e.g., CD209+ and plasmacytoid DCs) in TDLNs is ongoing and will further augment our understanding of the functional significance of DC clustering. Nevertheless, given this novel finding that increased DC clustering and maturation within TDLNs have a positive impact on clinical outcome in breast cancer patients, the next investigational steps should be aimed at devising methods to enhance DC clustering in vivo to generate a more robust anti-tumor response. There are reports where DC clustering and maturation have been successfully enhanced in vitro using various signaling molecules and cell surface receptor activation [34,35]. It would also be important to identify specific tumor-derived factors that impair clustering in vivo, and explore therapeutic strategies to block them. Understanding the underlying mechanisms of this novel discovery will help us induce a more robust DC response to tumor antigens, paving the way for more potent, efficacious anti-cancer DC vaccines.

## Conclusions

Previous studies from our group and others have shown that significant decreases in immune cell populations arise in TDLNs of breast cancer patients, and that these changes strongly correlate with clinical outcome. A novel hypothesis of this study is that not only numerical changes, but also spatial organization of DCs may impact immune function and clinical outcome in cancer. Using novel spatial analysis algorithms, we found that DCs in healthy lymph nodes aggregate into large clusters, predominantly composed of mature DCs. In contrast, DCs in breast cancer TDLNs (especially in tumor-invaded nodes) are either unclustered or organized into smaller clusters with fewer mature DCs. Importantly, the extent of DC clustering in tumor-invaded nodes correlated with the duration of disease-free survival in patients. Furthermore, DC clustering appeared to be an independent factor from DC maturity status, tumor characteristics, or treatment regimens.

To our knowledge, this is the first report to demonstrate that the spatial organization of DCs within TDLNs impact clinical outcome in human cancer. These novel results suggest that promoting DC maturation and clustering *in vivo* could enhance the efficacy of DC-based vaccines for cancer. Our work paves the way for further investigational studies into mechanisms and better immunotherapeutic strategies against cancer.

## Abbreviations

TDLN: Tumor draining lymph node; HLN: Healthy intramammary lymph node; NSLN-: Tumor free non-sentinel lymph node; NSLN+: Tumor invaded non-sentinel lymph node; DC: Dendritic cell; DBC: Density based clustering algorithm; IHC: Immunohistochemistry.

## Competing interests

The authors declare that they have no competing interests.

## Authors’ contributions

AYC, NB, and PPL conceived the study and drafted the manuscript; JM and DZC developed spatial analysis algorithms; AFS, GHL, SY, KH, and VCC carried out immunohistochemistal stainings of lymph node sections; DS, DNK, and ES recruited patients and collected clinical samples; AK developed the software for GemIdent and AYC, NB, and ZM conducted statistical analyses. All authors read and approved the final manuscript.

## Supplementary Material

Additional file 1 **The DBC algorithm used to define DC clusters.** Illustrating density-based clustering of DCs: Blue circles represent DCs, C: marks circumference of cluster, R: marks radius of cluster. Isolated DC refers to a DC not classified as clustered by the algorithm. Click here for file

Additional file 2 Clinical and therapeutic characteristics of survival analysis in patients by DC maturity. Click here for file

Additional file 3 Clinical and therapeutic characteristics of survival analysis in patients by DC clustering status. Click here for file
