# Supplementary material for: Spatial organization of dendritic cells within tumor draining lymph nodes impacts clinical outcome in breast cancer patients
Source: J Transl Med. 2013 Oct 2;11:242. doi: 10.1186/1479-5876-11-242 (PMC3852260; doi:10.1186/1479-5876-11-242)
Supplement: Additional file 1 — The DBC algorithm used to define DC clusters. Illustrating density-based clustering of DCs: Blue circles represent DCs, C: marks circumference of cluster, R: marks radius of cluster. Isolated DC refers to a DC not classified as clustered by the algorithm. [file 1479-5876-11-242-S1.pdf]

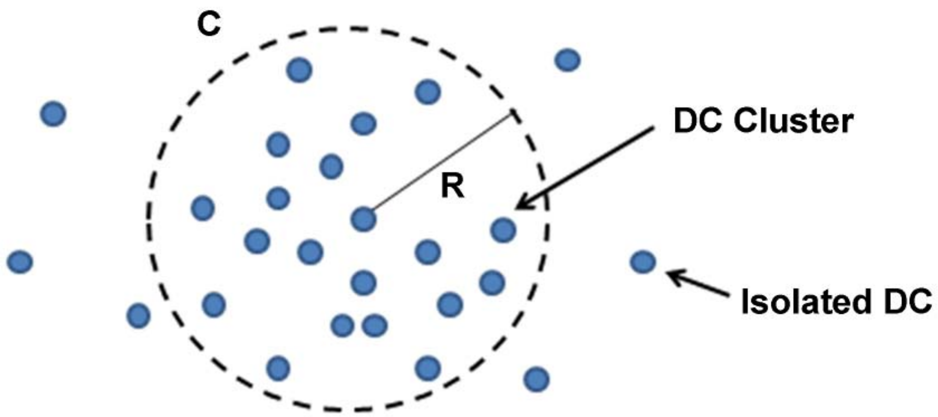

Additional File 1. The DBC algorithm used to define DC clusters. Illustrating density-based clustering of DCs: Blue circles represent DCs, C: marks circumference of cluster, R: marks radius of cluster. Isolated DC refers to a DC not classified as clustered by the algorithm.
